# Towards Better Understanding of COVID-19 Infection in Cancer Patients

**DOI:** 10.1093/jncics/pkaa091

**Published:** 2020-10-09

**Authors:** Marc S Hoffmann

**Affiliations:** Hematologic Malignancies and Cellular Therapeutics, University of Kansas Cancer Center, Kansas City, KS, USA

Keeping up with how coronavirus disease 2019 (COVID-19) should influence patient care challenges practicing hematologists and oncologists who routinely treat cancer patients. Many cancers unfortunately have an aggressive natural history that mandates therapeutic intervention on an urgent basis and does not afford us the luxury of postponing therapy while awaiting the end of a pandemic. As a consequence, our community was left with difficult decisions regarding how to care for our patients with a compelling risk of death from malignancy in the midst of a deadly infection whose biology was not fully understood.

As the initial data emerged from China in February and March 2020, it appeared that cancer patients were not only more susceptible to infection (1) but also had worse outcomes (2). In fact, the initial reports from Liang et al. (2) suggested that the risk of severe COVID-19 infection, a composite outcome of death or intensive care unit admission requiring mechanical ventilation, was 39% in cancer patients compared with 8% in patients without cancer. Although these initial reports were plagued by small sample sizes (n = 18 in the Liang study) (2), the data were compelling enough for many cancer centers to take added precaution with cancer patients. A comprehensive report from the American Society of Clinical Oncology released May 19, 2020, recommended a wide battery of precautions for cancer patients, including physical distancing measures such as limiting entry points, temperature-screening stations, universal personal protective equipment (PPE), and, importantly, testing of cancer patients before receiving immunosuppressive chemotherapy (3).

The current contribution from Basse et al. (4) included in this edition of *JNCI Cancer Spectrum* provides an important addition to our data set on cancer patients and offers a distinctly different view of how COVID-19 may affect cancer patients. The data comprise the largest longitudinally collected database from a single institution to date, with nearly 10 000 patients followed from March 1 through May 1 at the Institut Curie in Paris. Importantly, these data include both inpatients and outpatients as well as patients on active therapy and under surveillance and utilized a strict definition of PCR test and characteristic CT findings to establish confirmed cases. The population included a predominance of breast cancer (approximately 40% of the total cohort) in keeping with the overall institutional case mix.

Their results paint quite a different picture of COVID-19 risk among cancer patients. Not only was the overall incidence low (1.4%), it was comparable with or potentially lower than that in the French population at the time. Perhaps more strikingly, the mortality reported (19%) was identical to that in the French population at the time (20%). Finally, and this is likely the most important conclusion of the analysis, the risk of death or intensive care unit admission from COVID-19 on multivariate analysis was independent of cancer risk factors and wholly dependent on parameters of infection severity, namely presenting oxygen saturation and extent of lung involvement on CT.

Regardless of how one wishes to point out limitations, this report clearly is very good news for cancer patients, and suggests that their cancer diagnosis may not be as substantial a risk factor for severe COVID-19 infection as initially thought. Importantly, these results are in keeping with the prospective cohort of cancer patients in the United Kingdom (5). In that analysis, mortality was similar between cancer and noncancer patients, and disease-specific risk factors predicted mortality rather than cancer-specific risk factors, with advanced age conferring a nearly tenfold risk (5). These 2 studies offer a more complete and comprehensive view of how COVID-19 affects cancer patients.

So how should one incorporate this into clinical practice? First, it would be a mistake to conclude that aggressive infection control measures are no longer necessary. It is clearly in the best interest of our patients and society at large to limit COVID-19 infections as much as possible. Second, these studies are underrepresented for severely immunocompromised patients with hematologic malignancies undergoing intensive lymphodepleting chemotherapy or stem cell transplantation. These patients have more severe manifestations of community-acquired respiratory virus infections in general and tend to shed virus and remain infectious for longer periods of time (6). Extreme vigilance in the population clearly remains warranted. Third, these data can be helpful to alleviate anxiety and concern among our patients. Although the message clearly should not be to let their guard down, many cancer patients are already imprisoned by anxiety surrounding their cancer diagnosis. The social isolation and fear of death from COVID-19 infection compounds this anxiety. These data offer some hope to them that a COVID-19 diagnosis is not a death sentence. Finally, recommendations to test before delivery of lymphodepleting chemotherapy should remain in place. There are ample data that lymphopenia is associated with more severe COVID-19 infection (7), and, although this is observational data that do not prove causation, it is hard to imagine that lymphodepleting chemotherapy would be anything but possibly deleterious. The last word of caution is that one must be careful regarding each individual cancer drug and possible effects on COVID-19 infection severity. Many of us read with pleasure the report from Treon et al. (8) showing a possible protective effect of ibrutinib against severe COVID-19 infection. It is incumbent on the practicing oncologist to ensure understanding of how our drugs may affect COVID-19 prognosis, favorably or unfavorably, and counsel and test patients appropriately.

## Funding

There was no funding source.

## Notes

**Role of the funder:** Not applicable.

**Disclosures:** The author has no conflicts of interest to disclose.

**Author contributions**: The author is solely responsible for the content of this article.

## Data Availability

Not applicable.
